# Figure of Merit Enhancement of Laterally Vibrating RF-MEMS Resonators via Energy-Preserving Addendum Frame

**DOI:** 10.3390/mi13010105

**Published:** 2022-01-09

**Authors:** Temesgen Bailie Workie, Zhaohui Wu, Panliang Tang, Jingfu Bao, Ken-ya Hashimoto

**Affiliations:** 1 School of Electronic Science and Engineering, University of Electronic Science and Technology of China, Chengdu 611731, China; zhwu@std.uestc.edu.cn (Z.W.); mailfortpl@163.com (P.T.); k.hashimoto@ieee.org (K.-y.H.); 2 CETC No.26th Research Institute, Chongqing 400060, China

**Keywords:** acoustic resonators, effective electromechanical coupling coefficient, figure of merit, quality factor, insertion loss, RF-MEMS

## Abstract

This paper examines a new technique to improve the figure of merit of laterally vibrating RF-MEMS resonators through an energy-preserving suspended addendum frame structure using finite element analysis. The proposed suspended addendum frame on the sides of the resonant plate helps as a mechanical vibration isolator from the supporting substrate. This enables the resonator to have a low acoustic energy loss, resulting in a higher quality factor. The simulated attenuation characteristics of the suspended addendum frame are up to an order of magnitude larger than those achieved with the conventional structure. Even though the deployed technique does not have a significant impact on increasing the effective electromechanical coupling coefficient, due to a gigantic improvement in the unloaded quality factor, from 4106 to 51,136, the resonator with the suspended frame achieved an 11-folds improvement in the figure of merit compared to that of the conventional resonator. Moreover, the insertion loss was improved from 5 dB down to a value as low as 0.7 dB. Furthermore, a method of suppressing spurious mode is demonstrated to remove the one incurred by the reflected waves due to the proposed energy-preserving structure.

## 1. Introduction

RF-MEMS resonators have been used for many applications, ranging from acoustic filters used for communication systems to timing application, owing to their excellent power handling capability and low motional resistance [1,2,3,4,5]. These merits are associated with piezoelectric transduction, which offers strong electromechanical coupling, and the use of substrate materials that have a low intrinsic damping and high energy density, such as single-crystal silicon (SCS) [1].

Amidst the most attractive properties of RF-MEMS resonators are their compatibility with existing CMOS technology, making them highly attractive for monolithic integration with CMOS circuitry [6,7,8,9,10]. In most of their applications, resonators with a high Q are of great importance in fulfilling the stringent requirements of the device. Accordingly, in applications for oscillators, Q sets the phase noise performance [8,11]. In filters, a high Q reduces the insertion loss while narrowing the bandwidth [12,13,14], whereas, in sensing applications it benefits the sensing resolution [15,16,17]. However, in order to obtain the aforementioned requirements, the Q of piezoelectric resonators still needs to be improved. Another important parameter which expresses the electromechanical transduction efficiency of a resonator is the effective electromechanical coupling coefficient ($k_{eff}^{2}$) [18,19,20]. The product of these two parameters, as given in Equation (1) and used as a figure of merit (FoM), is an important parameter for judging how good a resonator is. For this, designing resonators with a higher figure of merit is a hot topic for research. Hence, numerous studies have made efforts to enhance the FoM of a resonator by improving one or both of Q and $k_{eff}^{2}$.

(1) $$FoM=Q\cdot k_{eff}^{2}$$

Even though there are many intrinsic and extrinsic loss mechanisms, such as dielectric loss, piezoelectric loss, surface loss, and Ohmic loss, it has been widely reported that anchor loss is the most dominant loss mechanism of MEMS resonators severely limiting the quality factor effect in laterally vibrating RF MEMS resonators [21,22,23,24,25,26]. This loss occurs due to the elastic wave propagating out from the resonant cavity to the supporting substrate through the supporting tether. The primary design approach to reduce this loss is to locate the tether at the nodal points of the resonator where there is a minimum vibration, reducing the width of the tether and using a tether length of quarter an acoustic wavelength [27,28,29,30]. However, a limit to reduce the tether dimension for practical feasibility is reported as the pitfall of these approaches. In Ref. [31], our group demonstrated the effect of swastika hole-shaped phononic crystals (SW-PnC) with significantly high acoustic band gaps; these, on their placement on the anchoring boundaries, improve the quality factor by over 1.4-fold. Furthermore, in [32] the effect of placing 2 D-PnC on the tether and/or the anchoring boundaries of the resonator is demonstrated, and an improvement of about 3-fold in Q is reported. Another approach reported recently is deploying acoustic reflectors on the anchoring boundaries of the resonator, aiming to reflect some of the radiated elastic waves back to the resonant plate [33]. The major pitfall of these techniques is the limited enhancement in Q, as the reflection of acoustic waves is finished once it reaches the anchoring boundaries or at least after it passes the supporting tether. Furthermore, a MEMS support design to reduce the anchor loss of PZT-on-Si disc resonators by using a mechanical impedance transformer design on the tether is reported in [34]; this achieved a significant improvement in reducing the anchor loss.

This paper aims to introduce a new approach for improving FoM by using a suspended energy-preserving addendum frame. We firstly describe the layout of suspended energy-preserving addendum frames along with the mechanical equivalent model and transmissibility analysis in comparison with that of conventional resonator structure. Subsequently, the design and simulation of resonators with the proposed energy-preserving features and the potential improvements in the Q are explored through a series of simulations. Afterwards, frequency domain analysis is performed for all resonators, and the electrical responses are thoroughly discussed, followed by a description of the spurious mode mitigation technique introduced in this study. The finite element package COMSOL Multiphysics was used to calculate the eigen modes and frequency responses of all the resonators designed. A loss factor ($\eta_{\varepsilon S}$) of 0.0003 was applied in all the simulations for the piezoelectric material (AlN) used for this work.

## 2. Energy-Preserving Suspended Addendum Frame

### 2.1. Mechanical Equivalent Model

**Figure 1:**
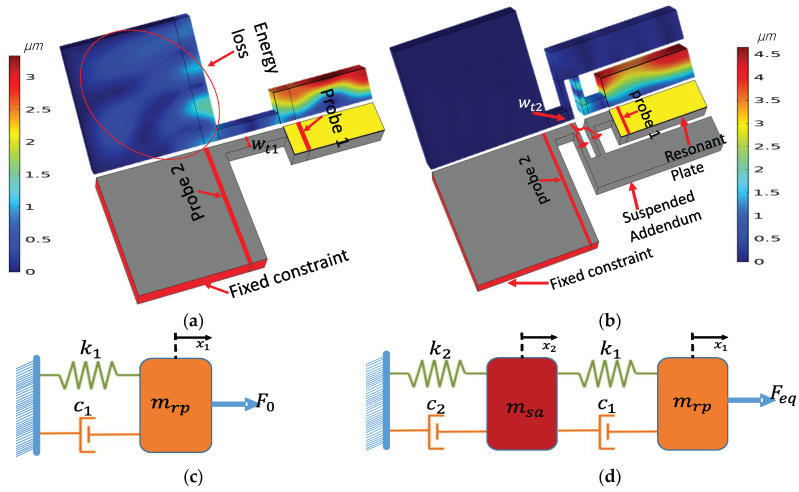
Illustration of: (**a**) Quarter plate conventional resonator used for the transmission test model along with its fundamental vibration mode shape at 140.32 MHz. (**b**) Quarter plate view of EPF resonator used for transmission test along with its fundamental vibration mode shape at 140.32 MHz. (**c**) Schematic view of a mass-spring-damper model used for the conventional MEMS resonator. (**d**) Schematic view of a mass-spring-damper model for a resonator with energy-preserving frames (EPF).

As is described prior, the main source of dissipation in RF-MEMS resonators is the propagation of acoustic energy to the anchoring boundaries through the supporting tether, which leads to a decrease in Q. Aiming to isolate the acoustic vibration between the resonant plate and the supporting substrate and taking the idea of mechanical vibration isolators as a motivation, a suspended addendum frame is added to the conventional structure along with a novel meandering tether support. Figure 1a,b show the quarter plate of the conventional MEMS resonator and the modified resonator with the suspended addendum frame, hereafter named the energy-preserving frame (EPF), designed to vibrate at a frequency of 140.32 MHZ in the first fundamental mode. Figure 1c,d show the schematic view of a mass-spring-damper equivalent model for the conventional and EPF MEMS resonators, respectively. The conventional resonator can be represented by a simple mass-spring-damper system of a single mass:

(2) $$m_{rp}\frac{\partial^{2}x_{1}}{\partial t^{2}}+c_{1}\frac{\partial x_{1}}{\partial t}+k_{1}x_{1}=F_{0}$$

where $m_{rp}$, $x_{1}$, $c_{1}$, $k_{1}$, and $F_{0}$ stand for the mass of the resonant plate, the total equivalent displacement, the damping coefficient, the stiffness, and the applied force, respectively. Assuming that the quality factor of the resonator is mainly dependent on the anchor loss, it is reasonable to neglect the effect of damping. In that case, the displacement $x_{1}$ can be expressed as:

(3) x1=F0k11−r2

where $r=\frac{\omega }{\omega_{n}}$ and ωn=k1mrp. The force transmitted to the fixed support can be expressed as:

(4) $$F_{T}=k_{1}x_{1}$$

As a result, the ratio of transmitted force to the excitation force is called transmissibility, *T*, which is expressed as:

(5) $$T=\left|\frac{F_{T}}{F_{0}}\right|=\left|\frac{1}{r^{2}-1}\right|$$

On the other hand, the resonator EPF can be represented by the coupling of two masses: one for the resonant plate ($m_{rp}$) and the other for the suspended addendum frame ($m_{sa}$).

(6) $$m_{rp}\frac{\partial^{2}x_{1}}{\partial t^{2}}+c_{1}\left(\frac{\partial x_{1}}{\partial t}-\frac{\partial x_{2}}{\partial t}\right)+k_{1}\left(x_{1}-x_{2}\right)=F_{eq}$$

(7) $$m_{sa}\frac{\partial^{2}x_{2}}{\partial t^{2}}+c_{2}\frac{\partial x_{2}}{\partial t}+k_{2}x_{2}-c_{1}\left(\frac{\partial x_{1}}{\partial t}-\frac{\partial x_{2}}{\partial t}\right)-k_{1}\left(x_{1}-x_{2}\right)=0$$

where $x_{i}$, $k_{i}$, and $c_{i}$ stand for the displacement, stiffness, and damping of conventional and EPF resonators. With the same context to the single mass system, neglecting the damping effect, the motions of the masses $m_{rp}$ and $m_{sa}$ to an input force $F_{eq}$ are:

(8) $$x_{1}=\frac{(k_{1}+k_{2}-m_{sa}\omega^{2})F_{eq}}{(k_{1}-m_{rp}\omega^{2})(k_{1}+k_{2}-m_{sa}\omega^{2})-k_{1}{}^{2}}$$

(9) $$x_{2}=\frac{k_{1}F_{eq}}{(k_{1}-m_{rp}\omega^{2})(k_{1}+k_{2}-m_{sa}\omega^{2})-k_{1}{}^{2}}$$

The transmissibility of the energy isolator can then be expressed as:

(10) $$T=\left|\frac{F_{T}}{F_{eq}}\right|=\left|\frac{k_{2}x_{2}}{F}\right|=\left|\frac{-k_{1}k_{2}}{(k_{1}-m_{rp}\omega^{2})(k_{1}+k_{2}-m_{sa}\omega^{2})-k_{1}{}^{2}}\right|$$

Taking the law of conservation of energy into consideration and assuming that excitation forces are equal ($F_{0}=F_{eq}$) and of an equal stiffness ($k_{1}=k_{2}$), as the vibration of the suspended mass is caused by the resonant body, the displacement $x_{2}$ is smaller than $x_{1}$. As a result, the transmissibility of the resonator with a suspended frame is expected to be much lower than that of a conventional resonator (i.e., higher isolation capacity).

### 2.2. Transmissibility Analysis

To further demonstrate the acoustic wave attenuation capability of the proposed method, a transmission line simulation was performed for the EPF and conventional resonators. As can be seen in Figure 1a,b, an input frequency-dependent harmonic displacement excitation $U_{in}\left(f\right)$ with an amplitude of 1 nm was applied at the boundary probe 1. Then, the vibrational transmissibility could be sensed at boundary probe 2 as a harmonic displacement of $U_{out}\left(f\right)$. The frequency-dependent transmission (dB) could be obtained by the relation [30]:

(11) $$Transmission\;\left(dB\right)=20\log\left(\frac{U_{out}\left(f\right)}{U_{in}\left(f\right)}\right)$$

**Figure 2:**
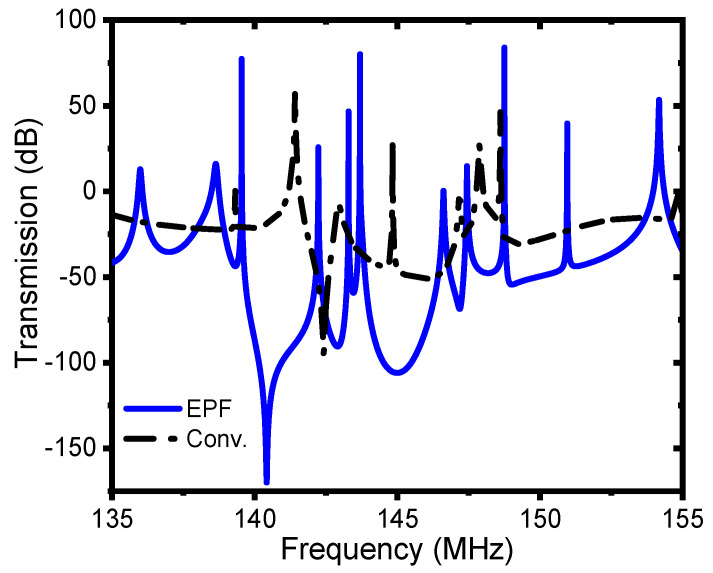
Transmission (dB) spectrum of the conventional and EPF resonators in the frequency range of 135 MHz to 155 MHz.

As can be seen from Figure 2, at the resonance frequency of 140.32 MHz the simulated attenuation for the conventional and proposed EPF resonators were 20 dB and 124 dB, respectively. This implies that the amount of energy transmitted to probe 2 (which is on the anchoring substrate of the resonators) for the conventional resonator was orders of magnitude greater than that of the EPF resonator. The large improvement in the attenuation of the EPF resonator was consistent with the displacement profile of the resonators in the anchoring boundaries seen in Figure 1a,b, among which the conventional resonator had a larger displacement, implying a larger amount of energy leaking from the resonant plate compared to the one with the EPF resonator.

## 3. Device Design and Simulation

**Figure 3:**
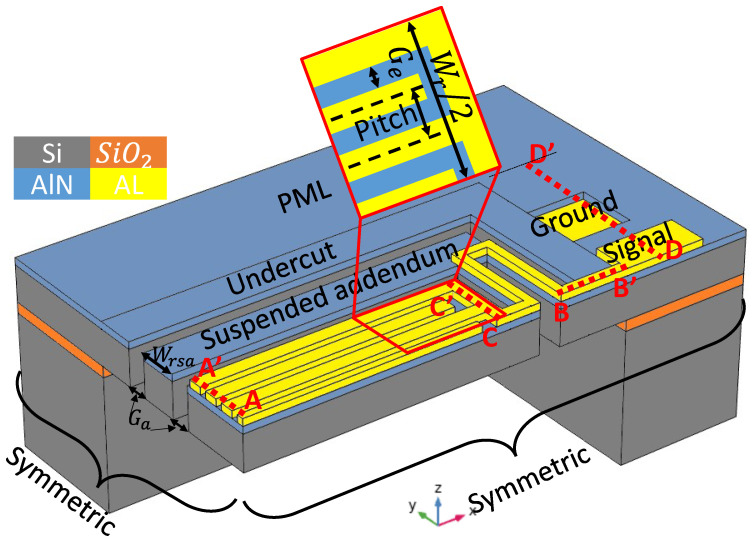
A mockup 3D view of a quarter plate resonator with an energy-preserving suspended addendum frame.

Cognizant to the resonators designed above for transmissibility analysis, all the resonators designed henceforth were set to vibrate in lateral mode with a frequency of about 140 MHz. The resonant plate was constructed from a piezoelectric film of aluminum nitride (AlN) sandwiched with a 10 µm anisotropic single-crystal silicon (SCS) substrate, which is also used as a common ground, and 1 µm-thick aluminum (Al) used as an interdigitated electrode for the input and output terminals. Furthermore, each of the resonators were designed to be transduced in the seventh order width-extensional (WE) mode. The seventh-order resonant frequency ($f_{r}$) of the width extension mode resonators was determined by the center-to-center distance of the electrodes (Pitch), which corresponds to 1/7 of the total resonant plate width ($W_{r}$), as can be seen from Figure 3. In other words, the *n*-th order resonant frequency ($f_{r}$) is *n* times the fundamental mode exhibited by the overall resonant plate.

(12) $$f_{r}=n\frac{\nu }{2w_{r}}$$

where *ν* is the velocity of the acoustic wave and *n* is the harmonic number that usually represents the number of IDT fingers (i.e., unless some portion of the resonant plate is left inactive—for example, such as reducing the loading effect of electrodes). Considering the much thicker single-crystal silicon used in this work compared to that of the piezoelectric material (AlN) and the electrode metal (Al), the vibration property of the device is mainly dominated by the single-crystal silicon. In this example, an acoustic velocity of 8500 m/s is used for analysis (i.e., acoustic velocity in anisotropic single-crystal silicon, <100> orientation). Despite the addition of some suspended structures (i.e., a frame) for the sake of improving the performance of the resonators, as is briefly discussed later, the dimensions of the resonant plates were consistent across all the resonators designed. In this regard, the length of the resonators ($L_{r}$), and the width of resonators ($W_{r}$), were designed with the proportion of 3.5 (i.e., $L_{r}$ = 3.5 × $W_{r}$).

It is noteworthy that, in order to reduce the computational time for the 3D FEA simulation of the resonators, only a quarter of the resonators were simulated and the symmetric condition was applied at the symmetric sides, as seen in Figure 3, to ensure that the eigen modes were solved for the whole resonant plate. Apart from this, a perfectly matched layer (PML) with a width of 3λ was applied to absorb the energy that escaped from the resonator and avoid reflections at the boundaries. Table 1 lists the values of basic design parameters for the resonators.

**Figure 4:**
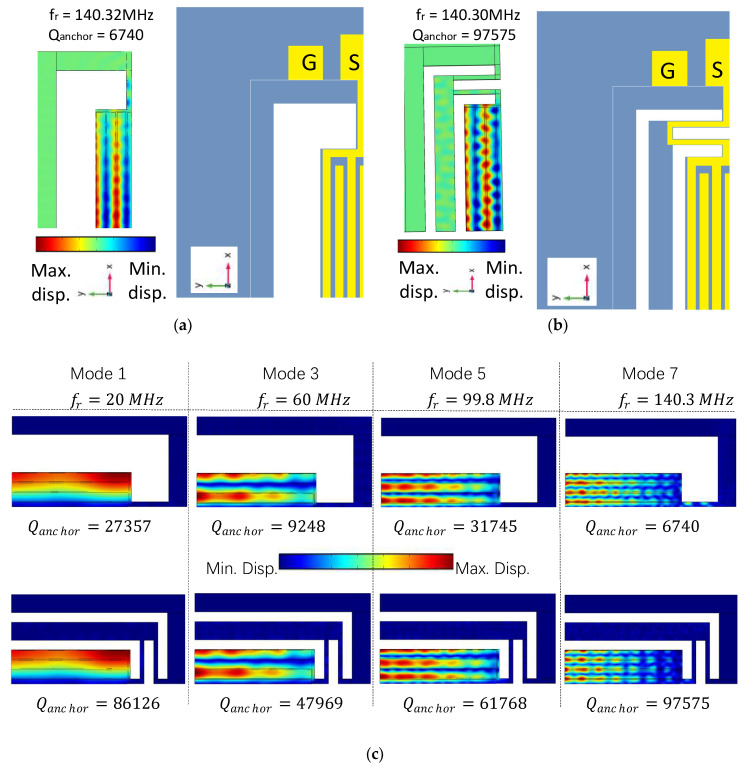
(**a**) Schematic top view along with the respective displacement profile (y-field) of the conventional resonator, (**b**) schematic top view along with the respective displacement profile (y-field) of the EPF resonator, and (**c**) FE simulated vibration modes (total displacement field) and their respective quality factors obtained using model analysis for conventional and EPF resonators in their fundamental (Mode 1), third-order overtone (Mode 3), fifth-order overtone (Mode 5), and seventh-order overtone (Mode 7) with a symmetric mode of vibration.

To fully demonstrate the ability of the proposed method to meet the stated objectives, two different devices were designed. Device one was a conventional resonator, as shown in Figure 4a, along with the corresponding displacement mode shape in the y-field. The second device was a resonator with a suspended energy-preserving addendum frame (EPF), as shown in Figure 4b. It can be seen in Figure 4a that the rectangular resonant plate of a conventional resonator was attached to the undercut by supporting tethers, which in turn were directly attached to the PML. Whereas, in the case of the EPF resonator, the resonant plate was not directly connected to the undercut. Rather, it was connected through the suspended addendum via a meandering tether with a width of ($W_{t2}$), which is half of the width of the tether ($W_{t1}$) (i.e., $W_{t1}=W_{t}/2$, Figure 1). The width of the addendum ($W_{sa}$) was tuned with FEA simulation, with an optimum value equal to two times the pitch width. Moreover, the suspended addendum frame was placed in an equal distance (i.e., gap of addendum ($G_{a}$)) from the resonant plate and the undercut.

Figure 4c presents the vibration modes of a resonator in its fundamental mode and three overtones (third, fifth, and seventh overtones) for the conventional and EPF resonators. For the sake of understanding the effect of vibration mode on the quality factor of the resonator, the same rectangular plate size was used. The fundamental mode along with three consecutive overtones with frequencies of 20 MHz, 60 MHz, 99.8 MHz, and 140.3 MHz, respectively, were analyzed with FEA modal analysis. Amongst the conventional resonators, the largest overtone in the list (seventh overtone) experienced the largest loss, resulting in the minimum $Q_{anchor}\;$(6740). Whereas, it was from the seventh overtone of the EPF resonator that the maximum $Q_{anchor}\;$(97,575) was achieved. Consequently, further analysis was conducted for this mode in order to find the impact of the proposed structure on the performance parameters.

From the mode shapes (displacement distribution and y-field) of the resonators presented in Figure 4a,b, it is apparent that the displacement in the supporting tether of the conventional resonator was much larger than that of the other three resonators, suggesting that the energy dissipation was higher (i.e., the color scale represents maximum displacement field with dark red and minimum displacement with dark blue).

**Figure 5:**
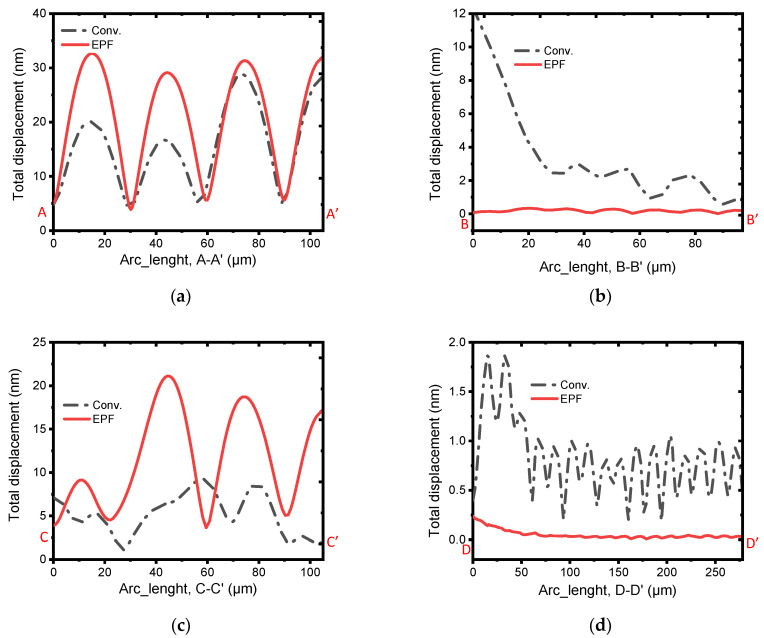
Illustration of the total displacement fields (μm) in the 7th-order width extension modes of the conventional and EPF resonators across the lines (**a**) A–A′, (**b**) B–B′, (**c**) C–C′, and (**d**) D–D′. The significantly higher vibration at the tether and the anchoring boundary of the conventional resonator compared to the others revealed the higher anchor loss endured by it.

Furthermore, the total displacement plots of the resonant plate at the A-A’ and C-C’ lines (see Figure 3), as demonstrated in Figure 5a,c, reveal that the conventional resonator had the minimum displacement. This implies that the amount of energy on the resonant body is lower than the EPF resonator. On the other hand, the plots shown in Figure 5b,d at the lines B-B’ and D-D’ (see Figure 3), which are at the tether and the anchoring substrate, respectively, revealed that the conventional resonator has significantly higher displacements. This indicates that the energy leaking into the supporting substrate is higher than that in the EPF resonators. On top of that, the FEA simulated $Q_{anchor}$ results of 97,575 for the EPF resonator were attained, which account for an improvement as high as 14.5 fold that of the conventional resonator’s result of 6740.

## 4. Discussion

**Figure 6:**
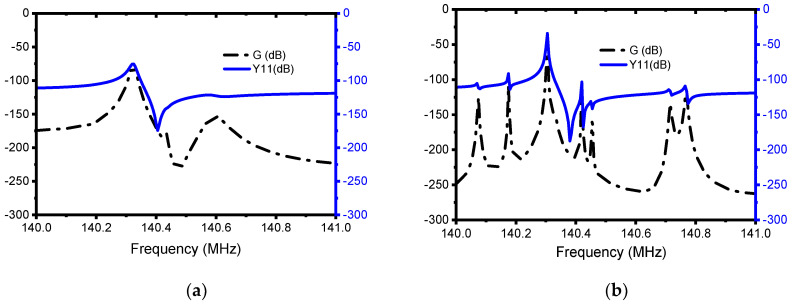
Illustration of the simulated admittance, Y11 (dB), and conductance, G (dB), response of the: (**a**) conventional resonator and (**b**) EPF resonator.

**Figure 7:**
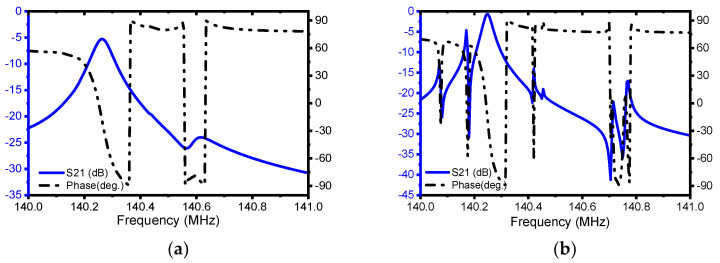
Illustration of the simulated S-parameter, S21 (dB) and phase (deg.) response of (**a**) the conventional resonator and (**b**) EPF resonator.

The FEA-simulated admittance response of the conventional resonator along with the EPF resonator is plotted in Figure 6a,b. The summaries of the results obtained are listed in Table 2, from which it can be observed that a $k_{eff}^{2}$ of 0.13% is obtained from the EPF resonator and one of 0.14% is obtained from the conventional resonator. This indicates that the employed techniques do not have a significant effect on the coupling coefficient. On the other hand, the simulated transmission S21 (dB) responses plotted in Figure 7a,b reveal a significant improvement in the insertion loss (IL) from 5 dB to 0.7 dB. The unloaded Q ($Q_{u}$) also shows a significant improvement of about 12.5-fold. On top of that, as shown in Table 2, the EPF resonator demonstrated a $Q_{u}*$ $k_{eff}^{2}$ product (i.e FoM) of 66) which shows an 11-fold improvement compared to the conventional resonator with a value of about 6.

The main pitfall encountered by the proposed FoM enhancement technique is that it introduces undesirable in-band and out-of-band spurious modes which mainly result from the reflected transverse waves. As these spurious modes highly affect the main mode, the study of the mitigation technique is necessary. In this regard, a new method of spurious reduction is introduced, as presented in the next section.

## 5. Spurious Mode Suppression

**Figure 8:**
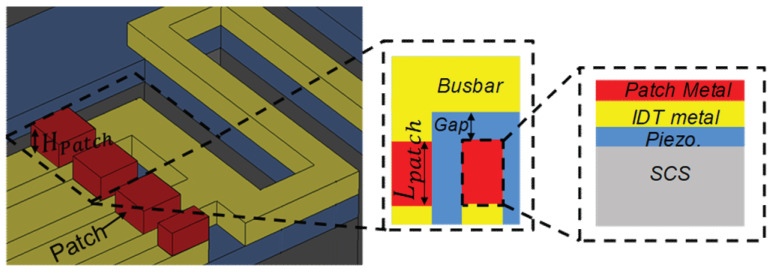
Schematic showing the proposed layout of metal patches on the edges of electrodes (inset: top view and side view of the selected regions at the edge of the IDT electrodes).

Spurious modes are considered as a major bottleneck in achieving high performance with RF-MEMS resonators. These unwanted spurious modes are mainly caused by reflections of acoustic waves by the acoustic boundaries and energy-preserving structures, in this particular case. As can be seen from the frequency response in Figure 6b, a significantly large amount of spurious mode appears before and after the main mode, which is not the case for the conventional resonator’s frequency response, as shown in Figure 6a. That being so, an approach for suppressing spurious mode is devised by adding metal patches on the edges of the electrodes, as is illustrated in Figure 8.

The metal patch on the edge of an electrode creates different acoustic impedance zones wherein the transverse spurious waves could be significantly influenced into having a vanishing coupling, resulting in spurious free responses. The metal patch installed has patch depth ($H_{patch}$) and patch length ($L_{patch}$) as indicated in Figure 8. Moreover, the patch width is fixed to be identical with that of the IDT finger (electrode) width ($W_{e}$) for the sake of simplicity and its insignificant effect on the results.

**Figure 9:**
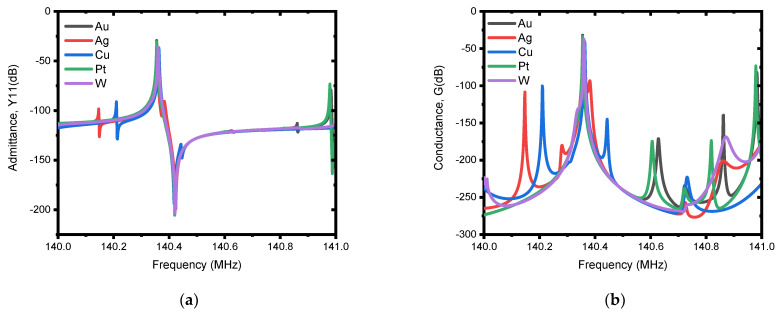
Illustration of simulated electrical responses with different patch metals (i.e., Au, Ag, Cu, Pt, and W). A patch length ($L_{patch}$) of $W_{e}/3$ and a patch depth ($H_{patch}$ ) of $H_{2}/m$ were used for the simulation. (**a**) Admittance, Y11 (dB), and (**b**) conductance, G(dB).

**Figure 10:**
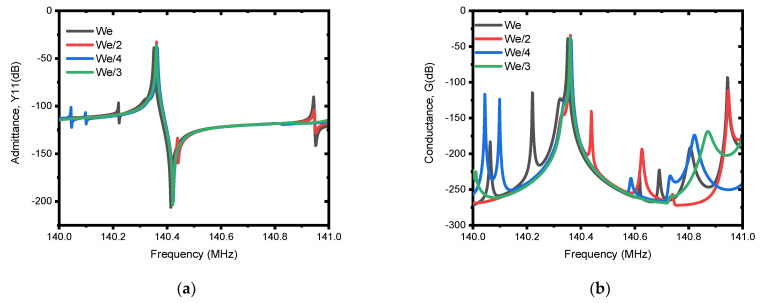
Illustration of simulated electrical responses using different patch lengths (i.e., $W_{e}/4$, $W_{e}/3$, $W_{e}/2$ and $W_{e}$). The patch depth ($H_{patch}$) of $H_{m}/2$ and patch metal of tungsten (W) is used for the simulation. (**a**) Admittance, Y11 (dB), and (**b**) conductance, G (dB).

**Figure 11:**
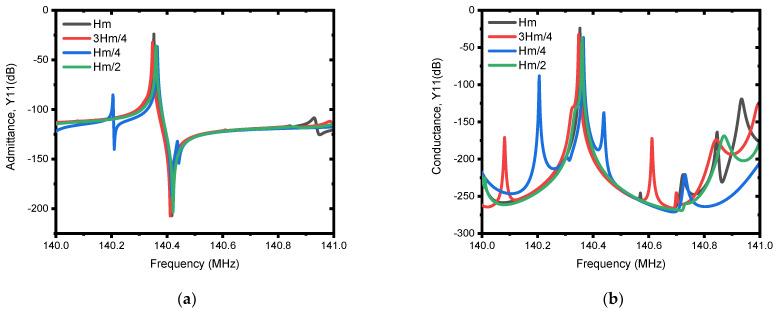
Illustration of simulated electrical responses using different patch depths (i.e., $H_{m}/4$, $H_{m}/2$, $3H_{m}/4$, and $H_{m}$). The patch depth ($L_{patch}$) of $W_{e}/3$ and the patch metal of tungsten (W) are used for the simulation. (**a**) Admittance, Y11 (dB), and (**b**) conductance, G (dB).

The first investigation is performed on the selection mechanism of materials to be used as a patch. In this regard, five metals (i.e., Ag, Cu, Au, Pt and W) were investigated. According to the resulting admittance, Y11 (dB), and conductance, G (dB), responses presented in Figure 9a,b, the performance is in direct correlation with the acoustic impedance (Z) of the patch metals used, which can also be seen from Table 3 and the frequency responses. Consequently, tungsten (W) shows superior performance, and is used in subsequent analysis to determine the other parameters of the patch. The optimum dimensions of the patch for the best suppression of spurious modes are tuned based on FEA simulations as it is demonstrated by the admittance, Y11 (dB) and conductance, G (dB) responses in Figure 10a,b and Figure 11a,b for different values of patch length and patch depth. From this, it can be observed that the responses for patch length of $W_{e}/3\;$and patch depth of $H_{m}/2$ demonstrated superior performance in offering spurious-free responses.

## 6. Conclusions

In conclusion, the proposed suspended energy-preserving addendum frame lends itself to acoustic energy confinement applications, as it offered a significant improvement in the FoM of about 11-fold compared to the conventional resonator. Using FEA-based modeling techniques, we found a design that led to an improvement in the unloaded quality factor ($Q_{u}$) as high as 51,136, approximately 12.5-fold that of the conventional resonator, which offered a maximum value of about 4106. The best insertion loss value recorded was 0.7 dB for the resonator with the proposed technique, which is significantly better than the 5 dB insertion loss value obtained for the conventional resonator. Aside from this, the proposed method does not have a significant effect on the effective electromechanical coupling coefficient. Moreover, a new method for suppressing spurious modes is introduced, from which the FEA simulation results demonstrate a superior performance in mitigating the spurious modes.

## Figures and Tables

**Table 1:** Resonator design parameters.

Symbol	Dimension	Value (µm)
$W_{r}$	Width of resonator	211.4
$L_{r}$	Length of resonator	740
$H_{Si}$	Substrate thickness	10
$H_{AlN}$	Piezoelectric material thickness	0.1
$H_{m}$	Electrode thickness	1
pitch	Electrode pitch width	30.2
$W_{e}$	Width of IDT fingers	26.2
$L_{t}$	Length of tether	120
$W_{t}$	Width of tether	40
$W_{u}$	Width of undercut	60.4
$W_{Sa}$	Width of suspended addendum	60.4
$W_{t2}$	Width of meandering tether	10
$G_{a}$	Gap of addendums	42

**Table 2:** Summary of the simulated results.

Device	IL (dB)	$Q_{l}$	$Q_{u}$	$k_{eff}^{2}\;(\%)$	FoM
Conventional	5.28	1870	4106	0.14	6
EPF	0.7	4007	51,136	0.13	66

$Q_{l}=\frac{f_{s}}{\Delta f_{\left(-3dB\right)}}$, $Q_{u}=\frac{Q_{l}}{1-10^{\left(-\frac{IL}{20}\right)}}$, $k_{eff}^{2}=\frac{\pi^{2}}{8}\left(\frac{f_{s}{}^{2}-f_{p}{}^{2}}{f_{s}{}^{2}}\right)$, FoM= $Q_{u}*k_{eff}^{2}.$

**Table 3:** Properties of metals considered for the patch design.

Properties	Ag	Cu	Au	Pt	W
Density $(g/cm^{3}$)	10.5	8.94	19.32	21.4	18.7
Longitudinal velocity $\left(cm/s\right)\times 10^{2}$	3.2	4.65	3.24	3.96	5.23
Acoustic impedance $(g/cm^{2}\;s)\times 10^{5}$	37.8	41.55	62.6	84.74	97.86

Z=ρ∗ν, where Z, ρ and ν are the acoustic impedance, density, and acoustic velocity.
